# Global and regional prevalence and incidence of systemic lupus erythematosus in low-and-middle income countries: a systematic review and meta-analysis

**DOI:** 10.1007/s00296-022-05183-4

**Published:** 2022-08-25

**Authors:** Francis Fatoye, Tadesse Gebrye, Chidozie Mbada

**Affiliations:** Department of Health Professions, Manchester Metropolitan University, Birley Fields Campus, Brooks Building, 53 Bonsall Street, Manchester, M15 6GX UK

**Keywords:** Systemic lupus erythematosus, Systematic review, Incidence, Prevalence, LMICs

## Abstract

Systemic lupus erythematosus (SLE) may be more prevalent among most ethnic groups in the low-and-middle income countries (LMICs), still these countries are under-represented in epidemiological data on SLE. The aim of this study was to review the prevalence and incidence of SLE in LMICs and use meta-analytic techniques. The MEDLINE, CINHAL, Web of Science, Scopus and Global Index Medicus databases were searched for relevant studies published up to July of 2022. Papers selected for full-text review were included in the systematic review if they provided the prevalence or incidence of SLE in LMICs and published in English language. The reference lists of included articles were also searched for additional studies. Two individuals independently performed abstract and full-text review, data extraction, and quality assessment of the papers. The prevalence and incidence of SLE were pooled through random effects model. Pooled estimates were expressed with 95% confidence. Out of 2340 papers, 23 studies were included in the review. The mean age at diagnosis ranged from 25.5 to 45.8 years. Three studies were conducted in Argentina and Brazil, two studies in China and one study in Cuba, Colombia, Democratic Republic Congo, Ecuador, Egypt, India, Kenya, Malaysia, Mexico, Nigeria, Pakistan, Turkey, Ukraine, Venezuela, and Zimbabwe. The SLE prevalence and incidence varied from 3.2 to 159 per 100,000 and 0.3–8.7 per 100,000 persons, respectively. In a random effects meta-analysis (*n* = 10), the pooled prevalence of SLE was 103 (95% confidence interval [CI] – 17 to 224) per 100,000. Meta‐analysis of data from 6 incidence studies revealed an incidence of 5 cases per year (95% CI 2–8) per 100,000. According to WHO regions, the pooled prevalence of American and Western Pacific regions was 300 (95% CI – 200 to 900) and 36 (95% CI 35–37) per 100,000, respectively. The pooled incidence of the American region was 10 (95%, 0–14) per 100,000 inhabitants. Systemic lupus erythematosus is a common disease with considerable variation in prevalence and incidence among the general population in LMICs. Accurate estimates of prevalence and incidence of SLE are required to put in place appropriate programmes to reduce its burden in LMICs. ***PROSPERO registration number***: CRD: 42020197495, https://www.crd.york.ac.uk/prospero/.

## Introduction

Systemic lupus erythematosus (SLE) is a chronic autoimmune disease that causes inflammation of connective tissues [1]. In general, SLE has propensity to affect every organ and tissue of the body, and its pattern of clinical manifestations varies widely among patients [2]. The complex interactions among genetic disposition, environmental risk factors, and the hormonal status contribute to the clinical heterogeneity in clinical manifestation of SLE, thus making its often problematic or keenly dependent on clinical expertise, in addition to immunological findings [2–5].

The incidence and prevalence of SLE vary widely in population demographics, socioeconomic factors, and certain ethnic population, such as Hispanic population, black and Asian [1, 6–8]. Specifically, in Europe and North America, people of African descent [9, 10], American Indians and Alaska Natives [11, 12] have higher predilection and worse outcomes from SLE than Caucasians within the same contexts [13]. Therefore, there are numerous indications that SLE is less severe in patients of European ancestry than Asian, African, and certain “Hispanic” or various indigenous populations [6, 14]. In Australia, Canada and USA, SLE disease among aboriginal/indigenous individuals are twofold to fourfold more common compared to non-aboriginal individuals [15]. Furthermore, patients from Asia and African ancestry are also likely to have a greater number of clinical manifestations, active SLE onset and higher mortality than white populations [16].

The clinical technicalities and complexity with diagnosing SLE may have contributed to assertions that the disease is infrequent in Africa [9, 10]. However, emerging reports indicates that the prevalence of SLE in sub-Saharan African was 1.7% (0.8–2.9 which is lower than the Asian–Pacific countries [19]. The overall incidence and prevalence of SLE across Asian–Pacific countries ranged from 0.9 to 3.1 and 4.3–45.3 per 100,000, respectively [18]. Furthermore, the incidence of SLE in North America and Europe ranged from 3.7 to 49 and 1.5 and 7.4 per 100,000 person-years, respectively [20–22]. Evidence also suggests that there is a gradual increase of SLE prevalence in North America, Europe and Asia [17]. Though, study design reporting bias, case definitions and SLE classification criteria may also result to a variation of the proportion of the population that has SLE [17].

Despite the variation of SLE across all age groups, it is more common between the ages of 15 and 45 years [23]. Evidence showed that in 10–20% of patients with SLE disease starts in childhood, this is due to increased renal, neuropsychiatric and cardiopulmonary disease [24–26]. The gender disparity of SLE is also widely recognised with a 1: 9 ratio of male to female patients. The incidence and prevalence of SLE in females is usually highest at 15–44 and 45–64 years of age, respectively [11].

Due to environmental, genetic, and racial factors the prevalence and incidence of SLE varies across the various regions of the world. For instance, changes in environmental factors are associated with increased in SLE [27]. Furthermore, the severity and course of SLE may often be related to the difference education, health insurance status, income level, ethnicity, medication compliance and level of social support. The survival rate of SLE patients in LMICs is lower than high income countries, this is due to higher mortality, poor intervention, and co-morbidities of infection [28]. To date there is no systematic review that summarized the prevalence and incidence of SLE published in LMICs [11, 21, 29]. The aim of this review was to summarise the global and regional prevalence and incidence of SLE in LMICs.

## Methods

This systematic review followed the recommendations of the Preferred Reporting Items for Systematic Reviews and Meta-Analysis (PRISMA) guideline [30]. The review was registered with PROSPERO—the International Prospective Register of Systematic Reviews CRD: 42020197495.

### Data sources and search strategy

An electronic database search was carried out on titles and abstracts till 11th of July 2022. MEDLINE, CINHAL, Web of Science, Scopus and Global Index Medicus databases and were used to search the literature. Search terms used were: lupus, systemic lupus erythematosus, disseminated lupus erythematosus’, lupus erythematosus disseminates and libman-sacks disease, prevalence, incidence, epidemiology, and rheumatic disease (see Appendix for detailed search strategy). In addition to these databases, hand searches from the references of the included studies were also used. All references were downloaded to EndNote X8 and duplicates were removed.

### Study selection

One reviewer (TG) conducted the search. Two independent researchers (TG & FF) screened titles and abstracts. The potential eligible papers were retrieved, and two reviewers (TG & FF) agreed the initial inclusion criteria. The inclusion criteria were population of all age groups with lupus, both retrospective and prospective study designs and prevalence and incidence estimates of lupus in LMICs and studies should be available in English and full text. LMIC classification was confirmed by cross-referencing with the World Bank list of Countries by Income Level. Conference proceedings, review articles, articles in press, abstracts or editorials were the exclusion criteria. Articles on SLE cases in the context of overlap syndrome were also excluded from the review. The classification for SLE was based on the 1982 American College of Rheumatology and/or revised 1997 American College of Rheumatology criteria [11, 31]. Any disagreement in study selection was resolved through discussion and consultation with a third reviewer (CM) where necessary.

### Data extraction

Data were extracted from the full text articles by one (TG) of the authors using a predefined form. The data extracted included authors and date of the study, population and setting, ethnicity/race, case definition, incidence rate and prevalence. The extracted data were cross-checked by another reviewer (FF). If disagreement occurred, consensus was reached through discussion.

### Appraisal of individual study quality

Two independent reviewers (TG & FF) appraised the quality of the included studies. The included studies were assessed using a risk of bias tool developed by Hoy et al. [32]. The assessment tool consists of 10 items addressing the external and internal validity. This tool consists of ten items, including six items addressing internal validity (i.e., measurement reliability) and four items addressing external validity (i.e., representativeness of sample). The questions can be responded as ‘yes’ or ‘no’. The overall risk of bias for each study was evaluated low, moderate, and low. Studies with scores of 9 or 10 ‘yes’-answers were considered to have low risk of bias; studies with scores of 7 or 8 were considered to have moderate risk of bias and studies with scores of 6 or less were considered to have high risk of bias. Any disagreement was resolved by discussion with the third author (CM).

### Data synthesis and analysis

Studies were included in the meta-analysis if they reported the number of SLE cases and sample denominator, the estimate with 95% CI, or the information with which to calculate the estimated prevalence or incidence. All studies reporting period prevalence were converted to annual prevalence estimate. A statistical heterogeneity was assessed with *I*^2^, a statistic that estimates the percentage of total variation due to heterogeneity across studies, where 0–25% was low, 26–74% moderate and 75% and over high statistical heterogeneity [33]. All the pooled estimates and 95% confidence intervals were calculated using a random-effects model. The Comprehensive Meta-Analysis software version 3 (CMA.V3) (Biostat, Inc., Englewood, NJ, USA) were used to analyse the data [34, 35].

## Results

### Study selection and characteristics

**Fig. 1:**
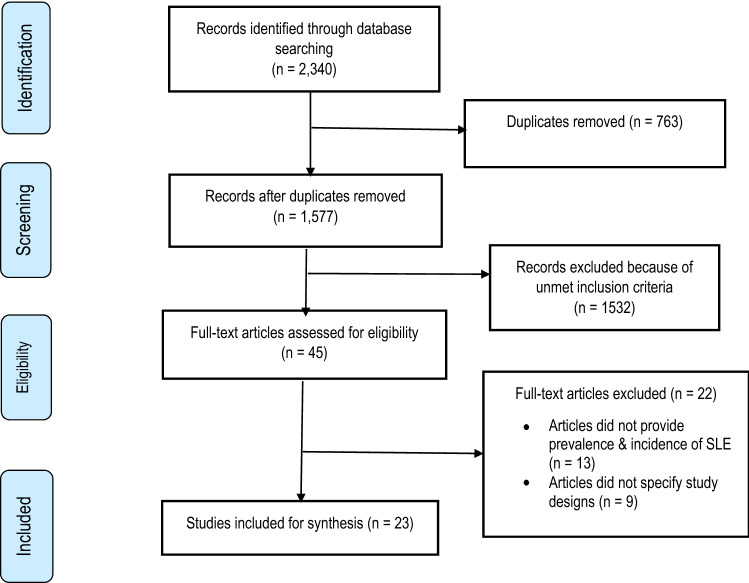
Flow diagram of publications included and excluded in the review

The systematic literature search results are presented in Fig. 1. Out of 2,340 titles, 23 studies that summarise the prevalence and incidence of SLE in LMICs were included in the review. Of the 23 included papers, 16 had low risk of bias and the remaining 7 had moderate risk of bias. The full risk-of-bias assessment is shown in Table 1.

**Table 1:** Basic characteristics of the studies retained for the analyses

Study, country	Study population (Mean age, years)	WHO regions	Sample size	Ethnicity/Race (%)	Case definition	Prevalence/ 100,000	Incidence/ 100,000	Risk of bias
Gonzalez et al. 2016 [36]; Argentina	Tucuma´n population; (30.5 ± 11.7)	AMRO	904,188 *W* = 93.5% *M* = 6.5%	83 Mestizos and 17 were African–Latin American	ACR	24.3 (95% CI 22.6–28.8)	1.8 (95% CI 1–2.9) 4.2 (95% CI 2.9–5.8)	Low
Pamuk et al. 2016 [37], Turkey	Rural and urban population in Turkey; (38.5)	EURO	620,477 *W* = 306,036 *M* = 314,411	N/A	ICD-10 code	51.7 (95% CI 46–57.4)	4.44	Low
Fernández-Ávila et al. 2019 [38]; Colombia	Colombian population (Over 18)	AMRO	47,663,162 *W* = 89% *M* = 11%	N/A	Not explicitly addressed	126.3	N/A	Low
Zou et al. 2014 [39]; China	General residents in rural Anhui, China (All age groups)	WPRO	1,358,725 *M* = 51.2%	All Chinese	ACR	36.03 (95% CI 35.54, 36.51)	N/A	Low
Ekwom 2013 [40]; Kenya	Patients attended at Kenyatta National Hospital (34)	AFRO	394 *W* = 100%	Black Africans	ACR	3000 [95% 1800–5600]	N/A	Moderate
Gbané-Koné et al. 2015 [41]; Nigeria	Patients attended in rheumatology department’s; (35.76)	AFRO	18,076	Black Africans	ACR	640 [95% 500–800]	N/A	Moderate
Wang et al. 1997 [42]; Malaysia	Patients attended in hospital (25.5 ± 10.1)	WPRO	539 *W* = 93%; Men = 7%	Chinese = 76 Malays = 17 Indians = 7	ACR	43	N/A	Moderate
Farooqi and Gibson 1998 [43]; Pakistan	Population in Punjabis, Pakistan	EMRO	700	N/A	WHO–ILAR COPCORD study	50	N/A	Low
Taylor and Stein 1986 [44]; Zimbabwe	Patients attended Mpilo Hospital (28)	AFRO	31 *W* = 30; *M* = 1	Mestizos = 83	ACR revised criteria	N/A	0.3	Moderate
Nakashima et al. 2011 [45]; Brazil	Patients in Cascavel, state Paraná (41.5 ± 14.44)	AMRO	291,747 *W* = 149,790 *M* = 141,957	N/A	ACR	N/A	4.8	Low
Vilar et al. [46]; Brazil	Patients living in Natal (31.8)	AMRO	493 239 (*W* = 269 900; *M* = 223 339)	White = 77 Non-White = 23	ACR	N/A	8.7 (95% CI 6.3 – 11.7) W = 14.1(95% CI 10.0 – 19.3) M = 2.2 (95% CI 0.7 – 5.2)	Low
Scolnik et al. 2014 [47]; Argentina	Patients attended in hospital, Buenos Aires	AMRO	127,959	All Caucasian	ACR	58.6 (95% CI 46.1–73.5)	6.3 (95% CI 4.9—7.7)	Low
Nasonov et al. 2014 [48]; Ukraine	Population in Ukraine (37)	EURO	367,500 *M* = 163,538 *W* = 203,962	98.6 Caucasian; 1.14 Asian	ACR	14.9 (95% CI 10.9–19.9)	0.3 (95% CI 0.0–1.8)	Low
Senna et al. 2004 [49]; Brazil	Individuals from Montes Claros (37)	AMRO	3038	White = 38 Non-White = 62	ACR	98	N/A	Moderate
Granados et al. 2015 [50]; Venezuela	Urban community in Venezuela (43.7)	AMRO	3,973 *M* = 1,606 *W* = 2,367	All Las Cocuizas	ACR	70 (95% CI 10–200)	N/A	Moderate
Pelaez-Ballestas et al. 2011[51]; Mexico	Patients from México (42.8 (SD 17.9))	AMRO	4059	N/A	ACR	90 (95% 20–200)	N/A	Low
Li et al. 2012 [52]; China	Chinese population, (45.8)	WPRO	14,642	N/A	ACR	30 (95% CI 0–60)	N/A	Low
Malaviya et al. 1993 [53]; India	Northern Indian population	SEARO	91 888	N/A	ANA	3.2 (95% CI 0–6.86)	N/A	Low
Malemba and Mbuyi-Muamba 2008 [54]; DRC	Patients attending hospital (49.7 ± 13.1)	AFRO	2370 *W* = 55.3% *M* = 44.7%	Black Africans	ACR	100 [95% 60–1500]	N/A	Moderate
Reyes-Llerena et al. 2009 [56]; Cuba	Patients with musculoskeletal complaints	AMRO	3155 *M* = 1238; *W* = 1917	N/A	ACR	60 (95% CI 10–250)	N/A	Low
Gheita et al. 2021[57]; Egypt	Patients of SLE across the nation (32.4 (SD 10.1))	EMRO	3661 (*W* = 3296; *M* = 365)	N/A	N/A	6.1	N/A	Low
Guevara- Pacheco et al. 2016 [55]; Ecuador	Patients living Cuenca City, 42.8 (SD 18.8)	AMRO	4877 (*W* = 59.7%)	N/A	COPCORD questionnaire	60 (95% CI 10–100)	N/A	Low
Quintana et al. 2016 [58]; Argentina	Patients in Rosario City (35.3 (SD 13.9))	AMRO	1656 (*W* = 61.5%)	N/A	WHO ICD-10	60 (95% CI 1–300)	N/A	Low

*WPRO* Western Pacific, *AMRO* American region, *EMRO* Eastern Mediterranean region, *AFRO* African region, *ACR* American College of Rheumatology criteria, *N/A* Not available, *W* Women, *M* Men, *WHO-ILAR COPCORD* World Health Organization and the International League of Associations for Rheumatology The Community Oriented Program for Control of Rheumatic Diseases, *EURO* European, *ACR* American College of Rheumatology criteria, *ANA* Anti-Nuclear Antibody test, *DRC* Democratic Republic of the Congo, *COPCORD* Community-Oriented Program for the Control of Rheumatic Diseases

The characteristics of the included studies are presented in Table 1. The included studies were from European regions (*n* = 2), Western Pacific regions (*n* = 3), the American region (*n* = 11), Eastern Mediterranean region (*n* = 2), the African region (*n* = 4) and Southeast Asia region (*n* = 1). The included studies were carried out in 17 countries. The studies were contributed from Argentina (*n* = 3), Brazil (*n* = 3), China (*n* = 2), Colombia, Ecuador, Egypt, Turkey, India, Malaysia, Pakistan, Ukraine, Venezuela, Zimbabwe, Mexico, Democratic Republic Congo, Cuba and Nigeria and Kenya. The mean age at diagnosis ranged from 25.5 to 49.7 years.

### Prevalence and incidence

**Fig. 2:**
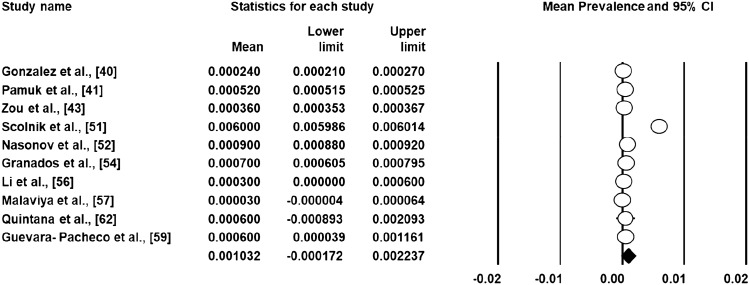
Prevalence of SLE across LMICs settings

**Fig. 3:**
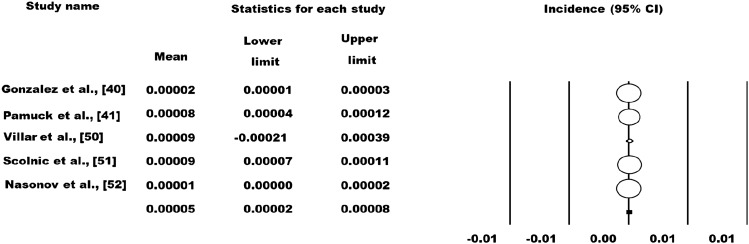
Incidence of SLE across LMICs settings. **a** Annual incidence of SLE, 2005–2012 in Tucuma´n, Argentina. **b** Incidence rates of SLE by 4-year periods (2003–2006, 2007–2010, 2011–2014**)** in Turkey. **c** Prevalence of SLE between 2012 and 2016 in Colombia

The prevalence and incidence rates of SLE varied from 3.2 to 3000 per 100,000 and 1.4–8.7 per 100,000 persons, respectively. The highest and lowest prevalence rate of SLE were reported in Colombia and Ukraine, respectively. In relation to the incidence rate the highest and lowest rate was recorded in Brazil and Ukraine. In the random effects meta-analysis (*n* = 10), the pooled prevalence of SLE was 103 (95% CI – 17 to 223) per 100,000 (Fig. 2). Meta‐analysis of data from five incidence studies revealed an incidence of 5 cases per year (95% CI: 2–8) per 100,000 (Fig. 3). According to WHO regions, the pooled prevalence of American region, Western Pacific regions and African region was 300 (95% CI – 200 to 900); 36 (95% CI 35–37) and 60 (95% CI – 40 to 1300) per 100,000, respectively. The pooled incidence of the American region was 10 (95% CI 0–14) per 100,000 persons.

### Sex related prevalence and incidence

Out of the total, twelve of the included studies reported the sex related prevalence or incidence of SLE (Table 2). The prevalence and incidence of men ranged from 0 to 90 and 0.5 to 2.6 per 100, 000 inhabitants, respectively. For the female population, the prevalence and incidence of SLE ranged from 23.8 to 204.3 and 4.72 to 14.1 per 100, 000 persons, respectively. Overall, the prevalence and incidence of SLE among women are higher than men.

**Table 2:** Sex-related prevalence and incidence of SLE per 100,000

Study	Number (prevalence)		Number (incidence)	
	Men	Women	Men	Women
Pamuk et al. 2016 [36]	314,411 (7 (95% CI 4.1–9.9))	306,036 (97.7 (95% CI 86.6–108.8))	314,411 (0.64 (95% CI 0–1.52))	306,036 (8.4 (95% CI 5.2–11.6))
Fernández-Ávila et al. 2019 [38]	42 million (20.3)	5.2 million (204.3)	N/A	N/A
Zou et al. 2014 [39]	642,036 (6.17)	611,796 (67.78)	N/A	N/A
Nakashima et al. 2011 [45]	N/A	N/A	141,957 (0)	149,790 (9.3)
Vilar et al. 2011[46]	N/A	N/A	223,339 (2.2 (95% CI 0.7–5.2))	269,900 (14.1 (95% CI 10.0–19.3))
Gonzalez et al. 2016 [36]	N/A	940,404 (34.9 (95% CI 32.8–41.1)	N/A	940,404 (4.2 (95% CI 2.9–5.8))
Scolnik et al. 2014 [47]	12,795 (23 (CI 95% 11.9–40.1))	115,163 (83.2 (CI 95% 63.9–106.4))	12,795 (2.6 (CI 1.2–3.9))	115,163 (8.9 (CI 95% 6.6–11.2))
Nasonov et al. 2014 [48]	183,600 (3.7 (CI 1.2–8.7))	229,900 (23.8 (CI 17.0–32.4))	N/A	N/A
Senna et al. 2004 [49]	1109 (90 (CI 0.0–260))	1929 (110 (CI 0.0–240))	N/A	N/A
Li et al. 2012 [52]	5223 (0)	5333 (60 (95% CI 10, 170))	N/A	N/A
Quintana et al. 2016 [58]	0	90 (95% CI 2–500)	N/A	N/A
Gheita et al. 2021 [57]	365 (1.2)	3296 (11.3)	N/A	N/A

*N/A* Not available

**Fig. 4:**
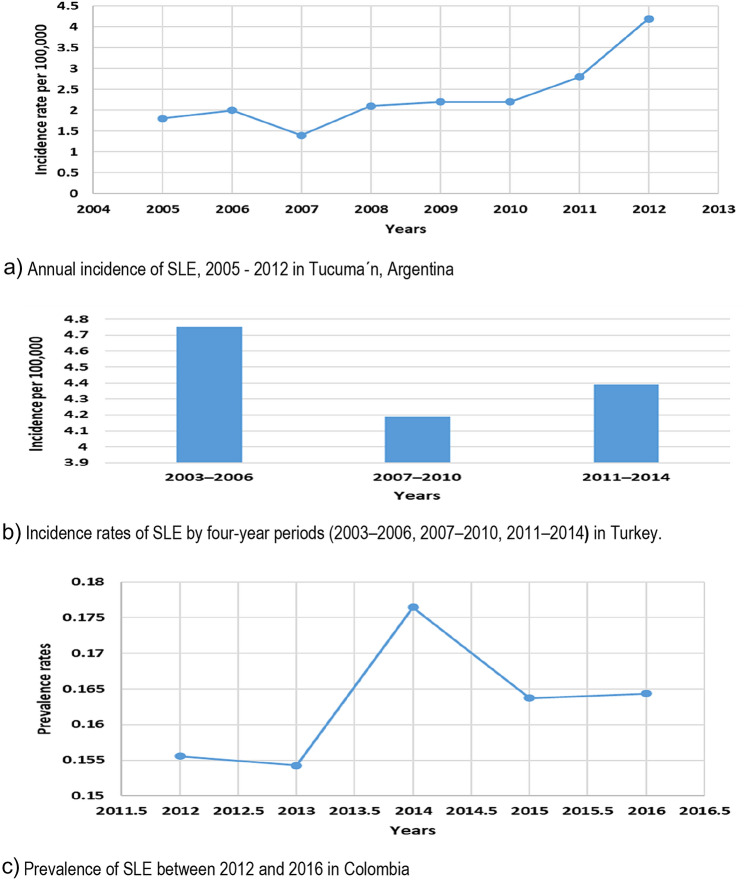
Incidence and prevalence of SLE stratified by years

Three studies were identified that examined the prevalence and incidence of people with SLE over time, allowing us to examine its temporal trend in Argentina [47], Turkey [37] and Colombia [38] (Fig. 4). The incidence of SLE in Argentina showed a substantial increase over the period of 8 years, the annual incidence in 2005 was 1.8 cases/100.000 inhabitants (95% CI 1–2.9) and in 2012 of 4.2 cases/100.000 inhabitants (95% CI 2.9–5.8). In Turkey [37], the annual incidence between the period of 2003–2006 (4.75 per 100,000 persons) were higher than 2011–2014 (4.39 cases per 100,000 persons). Number of patients with SLE also showed increment in Colombia from 15,556 to 16,437 per 100,000 persons for 2012 and 2016, respectively.

## Discussion

To our knowledge, this is the first review to evaluate and synthesise the incidence and prevalence data for SLE across the WHO regions of LMICs. This review represents a published data of prevalence and incidence of SLE from Argentina, China, Cuba, Colombia, Democratic Republic Congo, India, Kenya, Malaysia, Mexico, Nigeria, Pakistan, Turkey, Ukraine, Venezuela, and Zimbabwe. The prevalence and incidence of SLE ranged from 3.2 to 3000 per 100,000 and 0.3 to 8.7 per 100,000 persons, respectively. The highest estimates of incidence and prevalence of SLE were in Brazil [8.7(95% CI 6.3–11.7)/100 000 persons and in Kenya 3000 [95% 1800–5600]/100 000 persons, respectively. The lowest incidences of SLE were reported in Ukraine (0.3 (95% CI 0.0–1.8)/100 000 people, and the lowest prevalence was in India 3.2 (95% CI 0–6.86)/100,000 persons. Compared to high income countries, a lower prevalence of SLE is reported in LMICs. A systematic review on the incidence and prevalence of SLE reported that people in the in the United States of America (USA) 241/100 000 have much lower prevalence than people in Kenya 3000/100,000 [31]. On the other hand, evidence suggested that the highest incidence of SLE is reported in the USA (23.2/100 000 person-years) compared to Africa [1].

However, the results of the current review indicated that SLE is common in LMICs and the variation is considerably high across these countries. This variation is attributable to a variety of factors including the definition of SLE applied, ethnic and geographic differences in the populations being studied, access to health care, environmental (infections and ultraviolet light) and the methods of case identification [6, 59]. The incidence of SLE in Ukraine and Zimbabwe was the lowest compared to other countries in LMICs, this may be due to methodological differences used to collect the data. For example, the data sources and the skills used to identify SLE cases in Zimbabwe were inadequate, this is due to the data collected were relied upon hospital admission of one study hospital, and the low life expectancy of patients. On the other hand, the high prevalence estimate of SLE was reported in the Kenya, Nigeria, Colombia and Mexico, this may be because of an unadjusted rate in the population at risk.

Although the ratio varies, the prevalence and incidence estimate of SLE is more common in women than men. This may be due to oestrogen, a stimulant of lymphocytes, where women continue to have the higher oestrogen activity [60]. Moreover, clear differences in women and men immunity may also contribute to variation in response to predisposition of SLE [61, 62]. Most of the included studies in the current review reported a substantial difference of the prevalence and incidence of men and women. One study reported a prevalence rate of females (204) and male (20.3) per 100,000 persons [38]. The Incidence rate comparison between sexes also indicated this phenomenon, with one study contributing to the considerable variations of people with SLE in females compared to males [46].

Some studies that analysed the difference of occurrence of SLE among various ethnic groups in our review reported that black people to have high prevalence and incidence of SLE than white people [36, 46, 49]. It is also important to point out that there are studies that reported low incidence of SLE in black African population [43, 44]. The variation in the epidemiology of SLE is associated not only with genetic but also with environmental, sociodemographic, and sociocultural factors [61].

There are some strengths and limitations to be considered when interpreting the findings of this review. When identifying the relevant studies on prevalence and incidence of SLE, a systematic and rigorous approach was adopted. The adoption of different methodologies such as case identification method and analytical issues within the included studies have made it difficult to assess the epidemiological trend over time. Although an extensive literature search was carried out, we did not search for papers published in languages other than English, this could have influenced the incidence and prevalence estimates of SLE in LMICs. Due to the lack of financial resources and an inadequate health professional workforce, rheumatology services are limited or non-existent in many parts of Southeast Asia and sub-Saharan Africa [62, 63]. These may have resulted patients to a less likely to be diagnosed with SLE. The clinical and policy implications of this review is related with the female population who are at risk of SLE, it may help in the allocation of healthcare resources as well as priority for research funding. Moreover, the lack of epidemiological studies of SLE in Africa requires attention from researchers, and clinical and policy makers to understand its burden with the hope of improving the health outcomes of people with SLE in LMICs.

## Conclusions

Systemic lupus erythematosus is a common condition with considerable variation in prevalence and incidence among the general population in LMICs. The findings also suggested that the incidence and prevalence of SLE is higher in females compared with males. Furthermore, the trend of prevalence of SLE is increasing with time. The same to other public health problems, it is crucial for policy makers to intervene in the prevention and management of SLE. Furthermore, urgent need of research in terms of estimating its direct and indirect costs and impaired health related quality of life due to the condition is necessary in LMICs settings.
